# Supplementary material for: Risk Factors for Cytomegalovirus Infection and Its Impact on Survival after Living Donor Liver Transplantation in South Korea: A Nested Case-Control Study
Source: Pathogens. 2023 Mar 27;12(4):521. doi: 10.3390/pathogens12040521 (PMC10143532; doi:10.3390/pathogens12040521)
Supplement: Supplementary file 1 [file pathogens-12-00521-s001.zip › pathogens-2247146-supplementary.pdf]

Figure S1. Cumulative incidence of CMV infection within 12 months after LDLT.

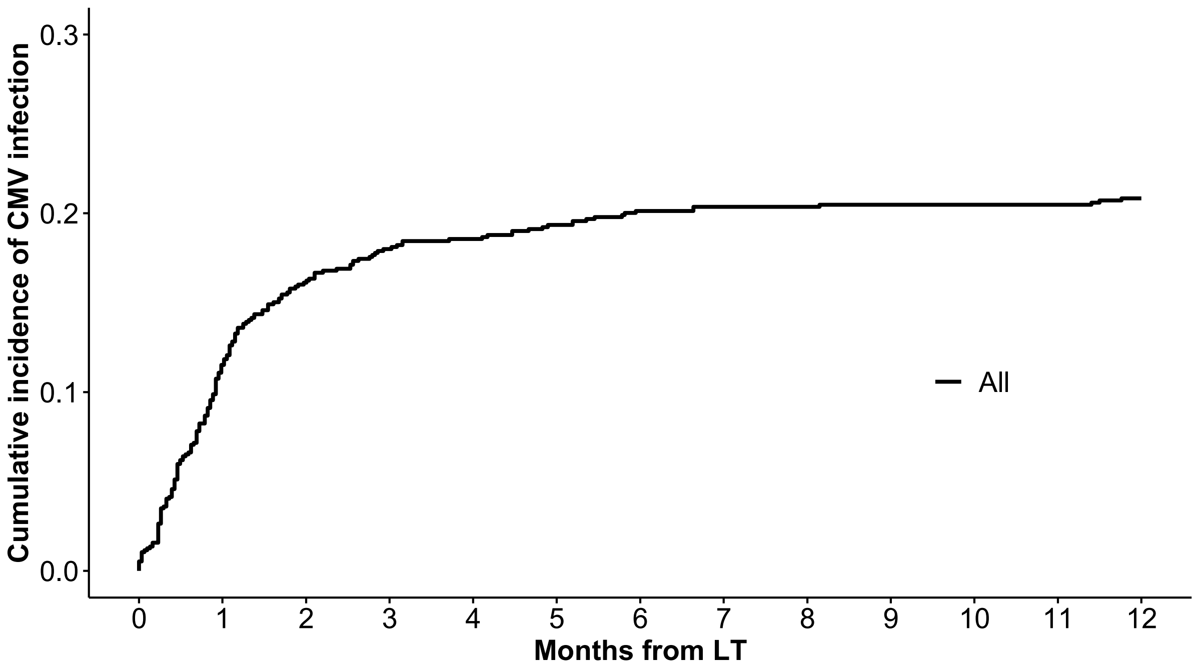

Number at risk

All	953	809	760	738	728	721	707	697	683	679	672	662	649

Figure S2. Density plot for days from LDLT to CMV infection.

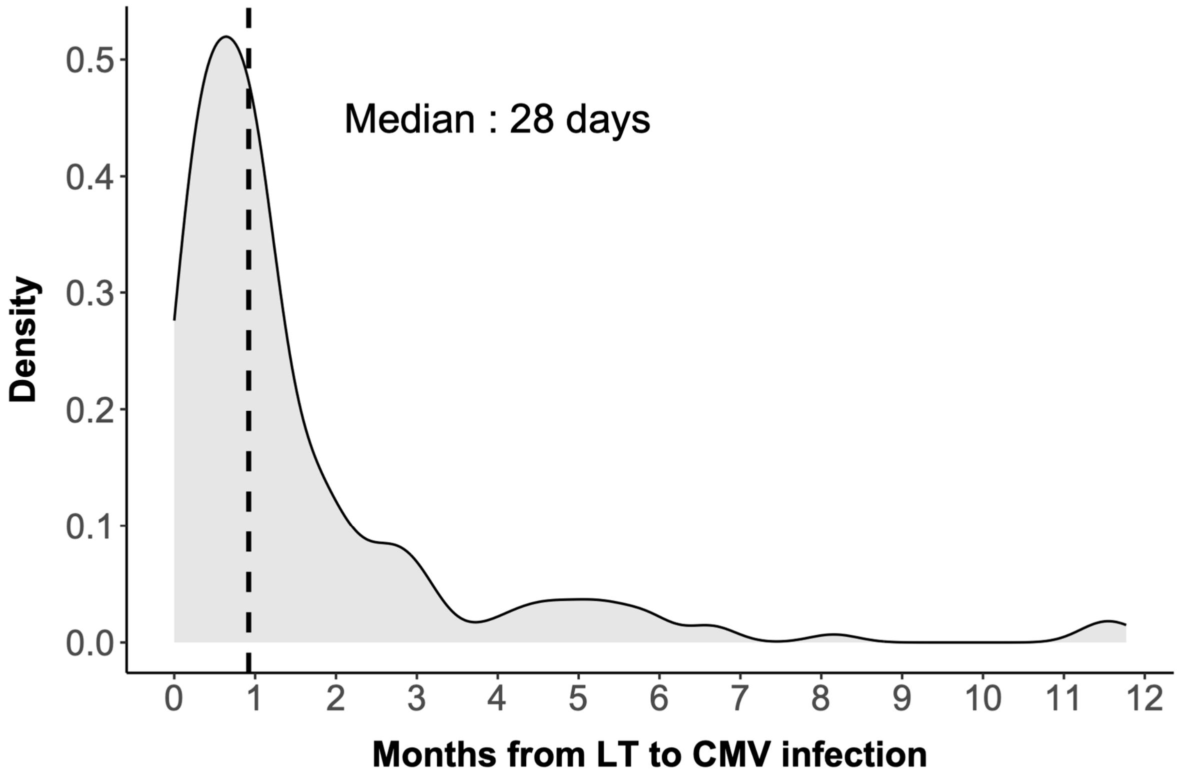

Table S1. Uni- and multivariable Cox regression for graft survival

Variables		HR (univariable)	HR (multivariable)
CMV_infection	No	-	-
	Yes	2.50 (1.58-3.95, p<0.001)	1.93 (1.16-3.20, p=0.012)
Age		1.01 (0.98-1.03, p=0.662)	-
Sex	Male	-	-
	Female	1.37 (0.84-2.22, p=0.206)	-
BMI		0.97 (0.90-1.04, p=0.402)	-
LT_year	2012-2015	-	-
	2016-2018	1.05 (0.62-1.81, p=0.846)	-
	2019-2021	1.35 (0.66-2.75, p=0.417)	-
Hypertension	No	-	-
	Yes	1.33 (0.79-2.25, p=0.281)	-
Diabetes_mellitus	No	-	-
	Yes	1.58 (0.99-2.52, p=0.056)	1.33 (0.82-2.16, p=0.251)
Cardiovascular_disease	No	-	-
	Yes	1.85 (0.85-4.03, p=0.124)	-
Underlying_liver_disease	Alcoholic	-	-
	Viral	0.97 (0.56-1.71, p=0.928)	-
	Others	0.96 (0.46-1.97, p=0.906)	-
HCC	No	-	-
	Yes	0.88 (0.55-1.39, p=0.572)	-
Pretransplant_MELD		1.04 (1.02-1.07, p=0.002)	1.02 (0.99-1.06, p=0.240)
Pretransplant_stay	OPD	-	-
	Ward	1.79 (1.12-2.85, p=0.015)	1.63 (0.99-2.67, p=0.055)
	ICU	3.42 (0.82-14.19, p=0.090)	1.48 (0.33-6.70, p=0.613)
Refractory_ascites	No	-	-
	Yes	1.33 (0.64-2.77, p=0.449)	-
Encephalopathy	No	-	-
	Yes	1.04 (0.53-2.02, p=0.919)	-
ABO_incompatibility	No	-	-
	Yes	1.32 (0.80-2.18, p=0.272)	-
Donor_age		1.02 (1.00-1.04, p=0.038)	1.02 (1.00-1.04, p=0.108)
Donor_sex	Male	-	-
	Female	1.20 (0.75-1.92, p=0.440)	-
Donor_BMI		1.04 (0.94-1.14, p=0.464)	-
Macrovesicular_steatosis	<10%	-	-
	>=10%	1.01 (0.56-1.84, p=0.968)	-
OP_time		1.00 (1.00-1.00, p=0.001)	1.00 (1.00-1.01, p=0.002)
Transfusion_RBC		1.03 (1.01-1.05, p=0.007)	0.99 (0.95-1.03, p=0.595)
Reoperation	No	-	-
	Yes	1.75 (0.84-3.65, p=0.136)	-
cr_preLT		1.21 (0.98-1.49, p=0.081)	1.10 (0.81-1.50, p=0.542)
albumin_preLT		1.44 (0.96-2.15, p=0.075)	3.08 (1.80-5.27, p<0.001)
glucose_preLT		1.00 (0.99-1.00, p=0.595)	-
hb_preLT		0.84 (0.75-0.94, p=0.003)	0.78 (0.67-0.90, p=0.001)
plt_preLT		1.00 (0.99-1.00, p=0.541)	-
wbc_preLT		1.01 (0.92-1.10, p=0.844)	-
neutro_preLT		1.01 (0.92-1.12, p=0.788)	-
lympho_preLT		0.62 (0.36-1.07, p=0.086)	0.76 (0.45-1.30, p=0.320)
TAC_mean		1.04 (0.98-1.11, p=0.225)	-
TAC_max		1.03 (1.00-1.06, p=0.091)	1.02 (0.99-1.06, p=0.160)
MMF_use	No	-	-
	Yes	0.92 (0.57-1.47, p=0.725)	-
mTORi_use	No	-	-
	Yes	1.08 (0.50-2.36, p=0.847)	-

Steroid_use	No	-	-
	Yes	1.12 (0.35-3.56, p=0.846)	-

Table S2. Full results of univariable logistic regression for CMV infection

Variables	OR (95% CI)	P
Age	0.98 (0.95-1.00)	0.084
Sex, female	2.31 (1.46-3.67)	0.001
BMI	0.96 (0.89-1.02)	0.187
Year of LT (2012-2015)		
2016-2018	1.00 (0.60-1.67)	1.000
2019-2021	1.00 (0.53-1.89)	1.000
Hypertension	1.28 (0.77-2.13)	0.333
Diabetes mellitus	1.04 (0.66-1.62)	0.877
Cardiovascular disease	1.63 (0.70-3.68)	0.246
Pretransplant MELD	1.09 (1.06-1.12)	0.001
Pretransplant stay (out-patient day)		
Ward	2.15 (1.38-3.36)	0.001
Intensive care unit	10.97 (1.59-216.69)	0.034
Refractory ascites	0.78 (0.35-1.64)	0.532
Severe encephalopathy	2.16 (1.20-3.90)	0.010
ABO incompatibility	1.83 (1.14-2.92)	0.012
Donor age	1.01 (0.99-1.03)	0.470
Donor sex, female	0.84 (0.54-1.31)	0.453
Donor BMI	1.01 (0.92-1.10)	0.875
Macrovesicular steatosis (≥10%)	1.70 (1.00-2.90)	0.050
Operation time, min	1.00 (1.00-1.00)	0.535
Transfusion of RBC, pack	1.03 (1.00-1.07)	0.043
History of re-operation after LT	2.14 (1.03-4.47)	0.040
Creatinine, mg/dL	1.71 (1.16-2.78)	0.017
Albumin, g/dL	0.86 (0.59-1.26)	0.448
Glucose, mg/dL	1.00 (1.00-1.01)	0.077
Hemoglobin, g/dL	0.92 (0.83-1.01)	0.093
Platelet, $10^3/\mu L$	1.00 (1.00-1.00)	0.936
White blood cell, $10^3/\mu L$	1.18 (1.09-1.29)	0.001
Neutrophil, $10^3/\mu L$	1.24 (1.13-1.38)	0.001
Lymphocyte, $10^3/\mu L$	0.76 (0.48-1.16)	0.212
Mean TAC trough level, ng/dL	1.01 (0.94-1.08)	0.817
Maximum TAC trough level, ng/dL	1.01 (0.98-1.05)	0.397
Use of immunosuppressants		

TAC	2.02 (0.29-39.65)	0.533
Mycophenolate mofetil	0.98 (0.63-1.54)	0.940
mTOR inhibitor	0.78 (0.35-1.64)	0.532
Steroid	1.00 (0.38-2.93)	1.000
Acute cellular rejection	1.02 (0.63-1.64)	0.935
